# Clinical and VNG Features in Anterior Canal BPPV—An Analysis of 13 Cases

**DOI:** 10.3389/fneur.2021.618269

**Published:** 2021-03-10

**Authors:** Prateek Porwal, Ananthu V. R., Vishal Pawar, Srinivas Dorasala, Avinash Bijlani, Prem Nair, Ravi Nayar

**Affiliations:** ^1^Vertigo and Balance Clinic, Bangalore, India; ^2^Aster Clinic, Zen Cluster, Discovery Gardens, Dubai, United Arab Emirates; ^3^Ear, Nose and Throat (ENT) Department, Jawaharlal Nehru Medical College (JNMC), Belagavi, India; ^4^Madhaw Medical Center, New Delhi, India; ^5^Department of Speech Pathology and Audiology, Amrita Institute of Medical Sciences, Kochi, India; ^6^Centre of Academics Research, HCG, Bangalore, India

**Keywords:** Anterior canal BPPV, VNG, VNG features, Dix Hallpike test, McClure Pagnini, Yacovino maneuver, Down beating Nystagmus, Benign paroxysmal positional vertigo

## Abstract

**Objective:** To define diagnostic VNG features in anterior canal BPPV during positional testing (Dix-Hallpike, supine head hanging, and McClure Pagnini tests).

**Study Design:** A retrospective study of patients diagnosed with anterior canal BPPV across four referral centers in New Delhi, Kochi, Bangalore, and Dubai.

**Subjects and Methods:** Clinical records of 13 patients with AC BPPV out of 1,350 cases, during a 3-years period, were reviewed and analyzed by four specialists.

**Results:** Four patients had positional down beating nystagmus with symptoms of vertigo during the bilateral DHP maneuver. Seven cases had positional down beating nystagmus only on one side of DHP. Typical down beating nystagmus was seen in 10 out of 13 cases during the straight head hanging maneuver. Down beating torsional nystagmus was seen in 6 out of 13 cases. Down beating with horizontal nystagmus was seen in three cases (in DHP and MCP mainly) while pure down beating nystagmus during SHH was only seen in four cases.

**Conclusion:** We conclude that anterior canal BPPV is a rare but definite entity. It may not be apparent on positional testing the first time, so repeated testing may be needed. The most consistent diagnostic maneuver is SHH though there were patients in which findings could only be elicited using DHP testing. We recommend a testing protocol that includes DHP testing on both sides and SHH. MCP testing may also evoke DBN with or without the torsional component. Reversal of nystagmus on reversal of testing position is unusual but can occur. The Yacovino maneuver is effective in resolving AC BPPV. We also propose a hypothesis that explains why DHP testing is sensitive to AC BPPV on either side, whereas MCP lateral position on one side is only sensitive to AC BPPV on one side. We have explained a possible role for the McClure Pagnini test in side determination and therapeutic implications.

## Introduction

Benign positional paroxysmal vertigo (BPPV) is the most frequent vestibular disorder displaying a 10% incidence rate in the general population (1). Posterior-canal BPPV accounts for 80–90% of cases, while lateral-canal BPPV (LC-BPPV) occurs in 10–20% of patients. Anterior (superior) canal BPPV (AC BPPV) is very rare (1–2%) (2–4).

Posterior canal BPPV, and lateral canal BPPV are well-defined entities, and their diagnosis is based on the direction of the nystagmus elicited by head position change, which include up beating and torsional in posterior canal BPPV and horizontal in horizontal canal BPPV.

The diagnostic criteria for BPPV of the anterior semi-circular canal (AC BPPV) is less clearly defined. Even the existence of the AC BPPV has been questioned (2).

The presence of a downbeat nystagmus with or without a torsional component during positional testing is the only described feature of AC BPPV (5). There is a need to firmly establish the existence of, define the diagnostic criteria, and clarify the treatment for AC BPPV.

To this end, we undertook an analysis of 13 cases of AC BPPV with a view to identify reliable diagnostic criteria during positional tests like supine head hanging (SHH), Dix-Hallpike (DHP) and McClure Pagnini (MCP) tests for this rare form of BPPV.

## Materials and Methods

This study analyzed patients diagnosed with AC BPPV from among over 1,350 patients of vertigo, seen in four referral centers for vertigo and dizziness in New Delhi, Kochi, Bangalore, and Dubai, during a 3-years period from 2017 to 2020. The records, both clinical and VNG, were each reviewed by all four vertigo specialists equally, and only 13 cases of AC BPPV were confirmed.

The patients were tested as per protocol and treated in their respective centers, and consent for the use of the data for publication after suitable anonymization was obtained.

The patients' details and VNG findings were discussed jointly by the consultants via video conferencing at a meeting convened for this study.

All these patients had been referred for a neuro-vestibular opinion after excluding other medical and central causes, because of one or more of the following problems: vertigo, dizziness, or postural instability.

The clinical history was compatible with BPPV, i.e., describing brief episodes of rotational vertigo lasting for a few minutes, when turning in bed, lying down and looking up, bending forwards, or extending the head backwards.

The physical examination consisted of a basic clinical, neurological evaluation and otoneurological examination such as range of motion of eyes, Otoscopy, Romberg and Unterberger tests, and cranial nerve and cerebellar tests.

Videonystagmography was undertaken using the same device (Balance Eye ®) in all centers. Balance Eye is a binocular VNG system which is class 2A European CE certified. It has the provision for eye movement recording with vision allowed and denied. We evaluated oculomotor activities like saccades, smooth pursuit, gaze with and without fixation and responses to horizontal high frequency headshake, hyperventilation, lateral canal head impulse test, and positional tests.

To explain the movement of otoconial debris in the McClure Pagnini position, we used the three-dimensional (3D) study tool of the membranous labyrinth named BPPV viewer developed by Traboulsi and Teixido (6).

The diagnosis of BPPV was confirmed using the diagnostic criteria established by the (2). These include a history of recurrent transient positional dizziness/vertigo and an induced positional nystagmus by the Dix-Hallpike test (DHP), McClure Pagnini supine roll test (MCP) done bilaterally, and supine head hanging test (SHH) (5, 7, 8).

A diagnosis of AC BPPV was made if a short duration (less than a minute) of vertical down beating nystagmus (DBN) with or without a torsional component was observed during the DHP and/or SHH tests. We used the Barany society criteria (9) to record the type of torsional nystagmus. The nystagmus beats predominantly downward but with a small torsional component in which the upper pole of the eye beats toward the affected ear (9).

The AC BPPV was classified as Definite if the vertigo was resolved by a repositioning maneuver (Yacovino maneuver) (5) and Probable if it was refractory (5).

## Case Reports

### Case 1

A 73-year-old female complained of vertigo of a spinning nature, 2 min in duration, and was present only when changing head posture and bending forward for 3 months. It was not associated with any other symptoms like headache, phonophobia, and photophobia. It was relieved after vomiting. Her medical and audiological examination was normal for her age.

### VNG Examination

DBN was seen during the DHP on the right with extension, the DHP on the left was negative. Down beating nystagmus was seen on the right lateral MCP and supine head extension (90°) maneuver. She was treated with the Yacovino maneuver and her symptoms resolved instantly. We unfortunately lost this patient before follow-up.

### Case 2

A 67-year-old lady had complaints of a “spinning” sensation with vomiting over the last 3 months with reduced hearing and aural fullness of the right ear. Over the last year she had occasionally experienced tinnitus in both ears, with the right being more affected than the left ear. She also had hypertension and cervical spondylosis. Audiological evaluation showed mild sensorineural hearing loss in the right ear and normal hearing with a sensorineural component at high frequencies in the left ear. Cervical VEMP was normal for both ears. The medical and neurological examination was normal.

### VNG Findings

The saccades and smooth pursuit examination were normal. Down and right beating nystagmus was seen during the DHP on the left and right (left > right).

The Yacovino maneuver was done but the symptoms recurred after 5 days. We attempted the Yacovino maneuver again on a second visit, but she was not relieved of her symptoms. We performed the DHP maneuver and saw that DBN was more on the left than the right side on her third visit. We performed the Yacovino maneuver again with her head rotated to the right side by 30° to make the left anterior canal more vertical. She was relieved of vertigo sensation after that maneuver. The symptom-free follow-up period is now 3 months.

### Case 3

A 43-year-old male came in with a history of spinning vertigo lasting for less than a minute starting 2 weeks prior. The vertigo was triggered by positional changes like sitting up from the supine position and while turning in bed. The vertigo was associated with nausea. He denied any history of head injury.

His clinical examination was normal. On positional testing the DHP test on the left side elicited the symptoms of vertigo and down beating torsional (upper pole of eye beating to left) nystagmus. On getting up, the patient had symptoms of vertigo without any apparent nystagmus on VNG. The MCP maneuver was negative on the right side. On the left side, the patient had symptoms of vertigo and down beating nystagmus. The SHH maneuver elicited vertigo and down beating torsional (upper pole of eye beating to left) nystagmus. The patient underwent the Yacovino maneuver with apparent success and his symptoms resolved and have not recurred over the last 6 months.

### Case 4

A 65-year-old male presented with vertigo and neck pain starting 2 years prior. Vertigo episodes were brought on from getting up from bed and looking down. It lasted for a few seconds along with the sense of imbalance. Audiometric findings revealed bilateral moderate to severe high frequency sensorineural hearing loss.

### VNG Findings

During the DHP test, a few down beats were seen with extension on the right, while the left was negative for vertigo and nystagmus. On the right lateral during MCP down and left beating nystagmus was seen, while returning to supine position displayed only down beats.

The Yacovino maneuver was done on follow-up and he recovered from vertigo attacks. He has been symptom-free for the past 2 months.

### Case 5

A 56-year-old lady complained of vertigo especially when looking down and turning her head to either side 1 month prior. She also had left ear pain and tinnitus and had fallen once for which she was admitted. She was unable to do her daily chores of house cleaning and washing clothes. Her medical and neurological examination was normal, and apart from degenerative changes in the cervical spine, there were no comorbid conditions.

### VNG Findings

Left and down beating nystagmus was seen on right during the DHP test, which reversed to right and down on making her sit, left, and DBN was seen on the right lateral of the MCP with reversal on getting up. Disappearance of symptoms after the Yacovino maneuver led to the diagnosis of AC BPPV. The patient has been symptom-free for 6 weeks now.

### Case 6

A 55-year-old gentleman presented with a history of short episodes of spinning vertigo when lying down in bed and when turning over in bed for the last 4 months. The episodes were short, eventually he also felt dizziness while walking. Dizziness subsided within 10 days but after 3 months he again felt the same dizziness for which he came to our clinic.

### VNG Findings

Down beats with associated vertigo were seen on right DHP maneuver. Short-lived down beats were also seen on the SHH maneuver. He recovered from vertigo after the Yacovino maneuver and has been symptom free for 2 months.

### Case 7

A 43-year-old male came in with a history of spinning vertigo lasting for less than a minute during that day. The vertigo was triggered by positional changes like sitting up from the supine position. He denied any history of head injury. His clinical examination was normal.

### VNG Findings

The DHP maneuver on the right side elicited the symptoms of vertigo and down beating nystagmus. The torsional component was not apparent. There was no reversal of nystagmus on sitting up. The DHP on the left side and MCP maneuver were negative. The SHH maneuver elicited vertigo and DBN. The nystagmus duration was only for a few seconds and for 4–5 beats. The torsional component was not apparent. The patient underwent the Yacovino maneuver and has been symptom-free for over a month now.

### Case 8

A 36-year-old male came in with a history of spinning vertigo lasting for less than a minute after 1 week. The vertigo was triggered by positional changes like sitting up from the supine position. He had associated nausea and vomiting. He denied any history of head injury.

His clinical examination was normal. The DHP on both the sides was negative. The MCP maneuver on the right side was negative and on the left side it elicited torsional (upper pole beating to left) nystagmus. The SHH maneuver elicited vertigo and down beating torsional (upper pole beating to right) nystagmus. The patient underwent the Yacovino maneuver and has been symptom free for a period of 1 month.

### Case 9

A 36-year-old female came in with a history of spinning vertigo lasting for less than a minute starting 3 weeks earlier. The vertigo was triggered by positional changes like bending down and moving her head while walking. She had associated nausea and denied any history of head injury.

Her clinical examination was normal. The DHP on both sides was negative but while getting up from the right side the patient had sitting up vertigo without any nystagmus. The MCP maneuver on the right and left side was negative. The first SHH maneuver was negative (probably due to voluntary convergence) but on sitting up the patient had vertigo with up beating nystagmus for 3 s. The second SHH maneuver showed down beating torsional (upper pole beating to left) nystagmus. The patient underwent the Yacovino maneuver and has been symptom free for over 1 month now.

### Case 10

A 50-year-old female came in with a history of spinning vertigo lasting for less than a minute starting over 6 weeks prior. The vertigo was triggered by positional changes like turning to left in the supine position. She had associated nausea and vomiting during a few episodes.

Her clinical examination was normal. She underwent positional testing. The findings were as follows: the DHP test on both sides was positive and showed DBN without a torsional component. The MCP maneuver on the right side was negative and on the left side it elicited torsional (upper pole beating to left) nystagmus. The SHH maneuver elicited vertigo and down beating torsional (upper pole beating to left) nystagmus. The patient underwent the Yacovino maneuver but the symptoms recurred again after 15 days. She recovered after a third Yacovino maneuver. She has been asymptomatic since then.

### Case 11

A 46-year-old doctor came to our clinic with a history of spinning vertigo lasting for a few seconds for the last 5 days. The vertigo was triggered by positional changes like turning to either side in the supine position. It was also triggered by looking up and down. Neurological and other examinations were normal.

She underwent positional testing with the help of VNG. The findings were as follows: the DHP test on both sides was positive and showed DBN without a torsional component. On the right side, the DHP showed down and right beating nystagmus. The SHH maneuver elicited vertigo and down beating torsional (upper pole beating to left) nystagmus. The patient underwent the Yacovino maneuver but it failed. She recovered after a fourth Yacovino maneuver. She has been asymptomatic since then (Supplementary Videos 1, 2).

### Case 12

A 52-year-old lady came in with a history of spinning vertigo lasting for less than a minute which began that day. The vertigo was triggered by getting up from the supine position. She had associated nausea and vomiting. She denied any history of head injury.

On positional testing, the DHP test on the right side elicited vertigo with DBN. On getting up from the right side, the patient had sitting up vertigo without any nystagmus. DHP on the left side was negative for symptoms and nystagmus, but the patient had sitting up vertigo without any apparent nystagmus. The SHH maneuver elicited vertigo and down beating torsional (upper pole beating to right) nystagmus. The patient underwent the Yacovino maneuver which resolved the symptoms.

### Case 13

A 70-year-old gentleman came to our clinic with spinning vertigo which began 2 weeks prior. Duration was for a few seconds and it was associated with position change and looking up and down. He had a known case of hypertension and was taking regular medication.

### VNG Findings

**Figure 1:**
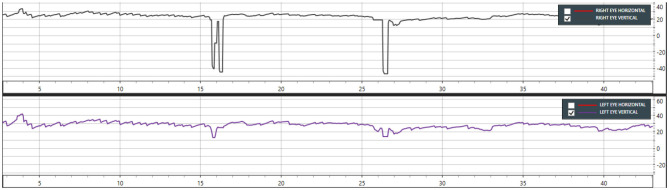
Case 13—Dix-Hallpike left showing down beating nystagmus from 5 to 40 s (Only vertical movements are shown in this graph).

**Figure 2:**
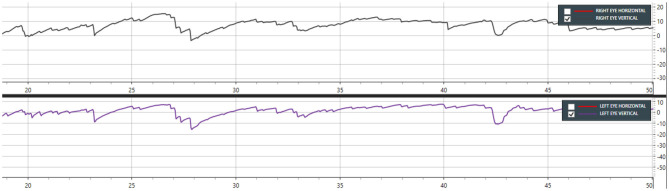
Case 13—Dix-Hallpike right showing down beating nystagmus from 19 to 48 s (Only vertical movements are shown in this graph).

**Figure 3:**
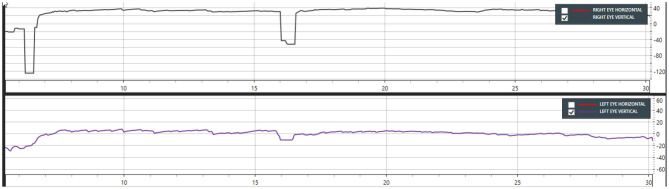
Case 13—Supine head hanging showing down beating nystagmus from 5 to 25 s. Note that DBN is most prominent in the left eye (Only vertical movements are shown in this graph).

DHP on both sides showed DBN. No nystagmus was seen on the MCP test. Down beating nystagmus was also seen during the SHH maneuver with extension. There was no reversal in any positional test. The patient underwent the Yacovino maneuver twice which resolved his symptoms (Figures 1–3).

Summary of all cases and findings are given in Tables 1, 2.

**Table 1:** Age, sex, history, and duration of vertigo in 13 patients.

**No**.	**Sex**	**Age**	**Clinical history**	**Head trauma**	**Time course**	**Sensorineural hearing loss (SNHL)**
1	F	73	Vertigo	No	3 months	No
2	F	67	HT	No	90 days	Mild SNHL R
3	M	43	Vertigo	No	14 days	No
4	M	65	Neck pain, vertigo	No	2 years	B/L mod SNHL
5	F	56	Ear fullness, tinnitus, and vertigo	No	30 days	Yes
6	M	55	Vertigo	No	120 days	No
7	M	43	Vertigo	No	1 day	No
8	M	36	Vertigo	No	7 days	No
9	F	36	Vertigo	No	21 days	No
10	F	50	Vertigo	No	6 weeks	No
11	F	46	Vertigo	No	5 days	No
12	F	52	Vertigo	No	1 day	No
13	M	70	Vertigo	No	14 days	No

**Table 2:** Findings in DHP, MCP, and SHH maneuvers with direction and type of nystagmus.

	**DHP right**	**DHP left**	**MCP right lateral**	**MCP left lateral**	**SHH/ Yacovino**	**Reversal**
1	Down	–	Down	–	Down	No
2	Down + H(Rt)	Down + H(Rt)	Not done	Not done	–	No
3	–	Down + T(Left)	–	Down	Down + T(Left)	No
4	Down	–	Down + H(Left)	–	Not done	No
5	Down + H(Left)	–	Down + H(Left)	–	–	Yes
6	Down	–	Not done	Not done	Down	No
7	Down	–	–	–	Down	No
8	–	–	–	T(left)	Down and T (Right)	No
9	–	–	–	–	Down + T(Left)	Yes
10	Down	Down	–	Torsional (Left)	Down +T(Left)	No
11	Down + T(Rt)	Down	Not done	Not done	Down + T(Right)	No
12	Down	–	Not done	Not done	Down + T(Right)	No
13	Down	Down	–	–	Down	No

*H(rt) and H(lf) indicates Horizontal right and left beating nystagmus. T(lf) and L(rt) indicates Torsional nystagmus with upper pole of eye beating to left and right side*.

## Discussion

The anterior semicircular canal is one of two semicircular canals in the sagittal plane, whose anatomical orientation is 41° to the sagittal plane while the posterior canal is oriented 56° to the sagittal plane. These canals converge at the crus commune, which leads to the utricle (10). Terms like superior canal and anterior canal are used interchangeably in literature. In this article we will use the term anterior canal (11).

Anterior canal BPPV (AC BPPV) is the rarest form of BPPV, with reported incidence of 1–2%, (4, 5, 12–14) although some authors have reported higher incidences in their series (10, 11). There is no consensus on the diagnosis and treatment, and the existence of AC BPPV as a separate entity has been questioned (15). In our study we noted an incidence of <1% (13 cases out of 1,350 cases of vertigo, pooled data from four specialized vertigo clinics).

Involvement of the anterior canal is suggested when a down beat nystagmus with a small torsional component appears on positional tests. The torsional component, if it is present, is usually in the direction of the involved ear as judged by the movement of the upper pole of the eye (12).

Note that DBN is also seen in cases of the apogeotropic variety of PC BPPV. DBN also occurs in central positional nystagmus associated with various brainstem and cerebellar lesions (16, 17), and this needs to be differentiated. In our study, central positional nystagmus was ruled out clinically and radiologically prior to evaluation in the vertigo and balance clinics in all cases.

There are two features that make determining the side of AC BPPV difficult. The first is that the torsional component may be very subtle and easy to miss. Second is that the nystagmus elicited is similar during DHP testing (down beating) on both sides even when the unilateral canal is involved, therefore the lateralization is hard to confirm (18–22).

According to the Barany Society criteria of 2015, the nystagmus of BPPV of the anterior canal can be elicited by any or all the positional tests, such as DHP tests unilaterally or even bilaterally and the SHH test (2) (Table 1).

Particles in the anterior canal can be free floating (canalolithiasis) or they might be attached to the cupula (cupulolithiasis) (23). Canalolithiasis of the anterior canal has a latency of up to 10 s and a maximum duration of 1 min. While cupulolithiasis of the anterior canal can present as persistent down beating nystagmus (more than 1 min) with or without torsional nystagmus (23).

### Dix-Hallpike Test (DHP)

The DHP test is traditionally considered as a test for BPPV of the ipsilateral posterior canal. However as the test aligns a canal pair, i.e., ipsilateral posterior canal and contralateral anterior canal along the gravity plane, it is actually a test of a pair of canals rather than an individual canal.

Note that during natural head movements, the same movement is stimulatory to one canal and inhibitory to the other canal in the pair. During DHP testing, the gravity-induced debris movement is stimulatory to the anterior canal when debris is present in the anterior canal and stimulatory to the posterior canal when debris is present in the posterior canal. Therefore, the elicited nystagmus is that of excitation of the involved canal.

### Down Beating Nystagmus

In our study, seven patients (1, 3, 4, 5, 6, 7, and 12) had positional DBN during the unilateral DHP test, while four patients out of 13 cases (cases 2, 10, 11, and 13) had positional DBN with symptoms of vertigo on the bilateral DHP test.

Casani et al. in their series of 18 cases noted positional DBN unilaterally with the DHP maneuver only in six cases and bilaterally in four patients (21).

Lopez-Escamez et al. found that DBN was seen during right DHP in five out of 14 cases, while during left DHP in only three cases. Three cases had positional DBN during both the left and right DHP test (22).

Similar findings are seen with Yang et al., where positional DBN was seen in 26 patients out of 40 during the DHP test (including both unilateral and bilateral), bilateral DHP was positive in 17 patients out of 40 (24).

Bertholon et al. reported that out of 12 patients, the nystagmus was triggered by the DHP test bilaterally in nine patients (75%) and unilaterally in one. In two patients with a typical history of positional vertigo but had a negative DHP test, the straight head-hanging maneuver was performed and was found to be effective.

The nystagmus of anterior canal BPPV is elicited in bilateral DHP testing even though only one side of the canal is involved (Eggers et al. and Barany's criteria) (19). It is believed that during a contralateral DHP maneuver, the affected canal is aligned with the gravity plane causing movement of the otolithic debris, whereas during an ipsilateral DHP test, the canal is oriented orthogonal to the gravity plane making it immune to gravity-induced debris movement. However, the fact that the anterior semicircular canal makes an angle of 40° with the para-sagittal plane and DHP testing involves turning the head by 45° results in the canal not being perfectly orthogonal to the gravity plane when performing the contralateral DHP test. This leads to movement of otolithic debris under the influence of gravity and explains the nystagmus.

### Torsional Nystagmus

In our study, torsional nystagmus was seen in four cases (2, 3, 5, and 11) during the DHP test.

According to Eggers et al., positional DBN of anterior canal BPPV is best seen in the SHH test (19). But the torsional component is usually better seen with DHP positioning and less frequently seen with a SHH test (4, 18, 21, 25).

Casani et al. noted that there was a torsional component of the nystagmus in six out of 18 cases, it was noted in five cases in unilateral DHP and in one case, they found it on bilateral DHP (21).

All 14 patients had positional DBN on different positional tests including DHP according to Lopez et al. (22). However, no torsional nystagmus was reported in their study (22).

According to Ewald et al. and von Brevern et al., the torsional component might or might not be seen in AC BPPV (2, 5, 13, 26), possibly because of the anterior canal's anatomical orientation which is closer to the sagittal plane (about 41°) compared to that of the posterior canal (56°) (16). However, the invariable demonstration of a torsional component during provocative testing of superior canal dehiscence syndrome (SCDS) argues against the anterior canal's anatomical orientation as an explanation for the lack of torsional component in BPPV.

Furthermore, the provocative test of SCDS is done in the upright position and positional testing moves the head to varying degrees of extension from the supine position. This gravity-related position difference during the observation of provoked nystagmus between the two conditions combined with the observation that the torsional component varies between DHP and SHH tests gives credence to our hypothesis that the difference in torsion is related to the difference in degree of otolithic influences on semicircular canal responses.

In our study, in two cases (8 and 9) no nystagmus was seen during the DHP test, of them nystagmus was seen during the SHH and MCP tests (case 8). We hypothesize that the degree of otolithic stimulation is the determinant factor in eliciting torsional nystagmus. The debris moving in the plane of the same canal causes different degrees of torsional nystagmus depending on the testing maneuver employed (DHP and SHH), and possibly reflects varying degrees of otolith semicircular interactions depending on head orientation with respect to gravity.

### Supine Head Hanging Test

In our study, 12 out of 13 patients underwent the SHH with extension maneuver and pure down beats were seen in four cases (cases 1, 6, 7, and 13), while down beating torsional nystagmus was seen in six cases in the SHH test.

### Down Beating Nystagmus in SHH

Three cases out of 14 had positional DBN on the SHH test according to Lopez et al. (22). According to Yang et al., during the SHH test, a DBN was observed in 33 (82.5%) patients (24).

### Torsional Nystagmus in SHH

In our study, down beating torsional nystagmus was seen in six out of 12 patients in the SHH test. Casani et al. noted that torsional nystagmus was seen in six out of 18 patients (21). Yang et al. noted that DBN with a torsional component was observed in seven patients out of 40 (24). Bertholon et al. reported that out of 12 patients, the DHP test was negative in two patients but the straight head-hanging (SHH) maneuver was positive. In six patients, torsional nystagmus was present during the positional tests (16).

In our study, we also found that SHH was more sensitive than the DHP test. It was positive in 10 cases out of 12. In two other cases (cases two and five), down beating nystagmus was only elicited during DHP tests.

### McClure Pagnini Test

The role of the MCP test in AC BPPV has not been clarified, but the presence of a horizontal component with DBN has been mentioned in a few studies (24, 27).

Imbaud et al. (27) in their series of 20 patients with AC BPPV saw horizontal torsional nystagmus beating toward the uppermost ear in the lateral supine position with reversal on standing.

In our study, down beating torsional nystagmus was seen in four cases in the DHP and MCP tests, also in some cases it was associated with a mild horizontal component with down beats. However, in a few positions of DHP and MCP, pure down beating nystagmus was also seen (cases 1, 4, 6, 7, 10, 12, and 13).

The vertical canal plane orientation is traditionally described as a tilt away from the para-sagittal plane toward the coronal plane. However, it is also known that the vertical canals are also tilted toward the horizontal plane. This results in the projection vector of rotations around the vertical canals influencing the projection vectors of rotations around horizontal canals resulting in the observed horizontal component of nystagmus.

In our study, an MCP test was done in nine cases. We found that the nystagmus was similar in directional properties to that seen in the DHP tests, though of a lesser intensity. In few cases, nystagmus was consistently unidirectional in DHP and MCP, if nystagmus was elicited on the right DHP then nystagmus was only seen on the right lateral of MCP. In case 3, the nystagmus was only seen in a left sided examination (left DHP and left lateral of MCP). While in cases 1, 4, and 5, nystagmus was only seen on the right side (right DHP and right lateral of MCP).

In case 10, down beating nystagmus was elicited on both DHP (right and left) but MCP was only positive on the left lateral, which was exactly same as the direction of torsional nystagmus and toward the side of canal involved.

**Figure 4:**
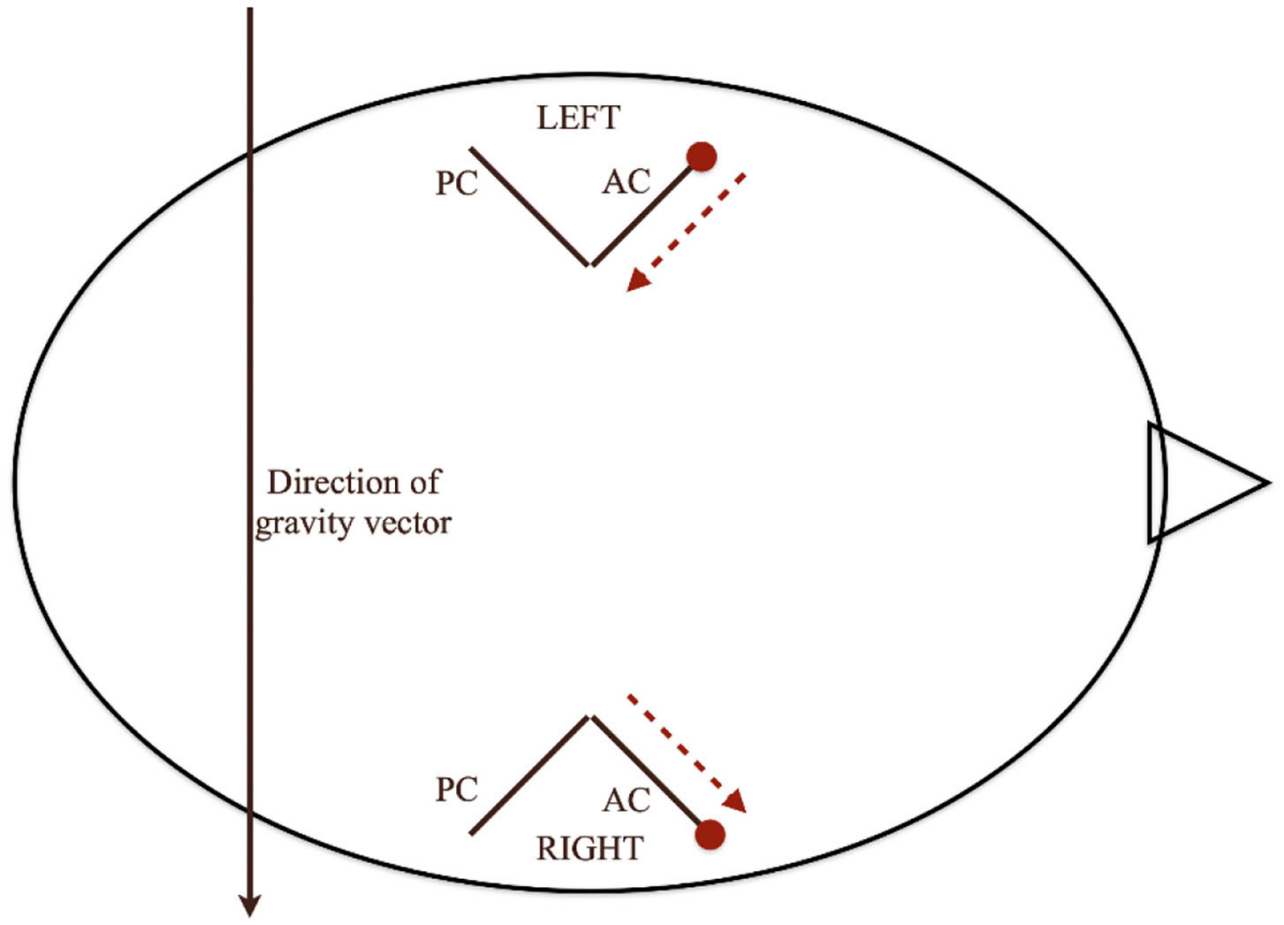
Movement of otoconia in MCP test. The direction of nystagmus during MCP may indicate the side of involvement. But during the DHP test on the right side or an MCP test in the right lateral position, the otoconial debris from the left anterior canal can be expected to move toward the common crus. However, no such movement of otoconial debris is expected in the right anterior canal as it is already in the most dependent position.

We propose that the direction of nystagmus on MCP may indicate the side of involvement. But on DHP tests on the right or an MCP in the right lateral position, the otoconial debris from the left anterior canal can be expected to move toward the common crus. However, no such movement of otoconial debris is expected in the right anterior canal as it is already in the most dependent position (Figure 4).

There can be three possibilities in the right lateral position of McClure Pagnini:

First, when the particles are at/near to the ampulla of the left (uppermost ear) anterior canal, no/minimal movement is seen in the right lateral position (part 1 of Supplementary Video 3). Second, when the particles are slightly away or distal from the ampulla (which might be due to DHP or SHH), we can see that the particles are moving toward the common crus (part 2 of Supplementary Video 3). Third, when the particles are in the right (undermost ear) anterior canal, in the right lateral position there is minimal or no movement of otoliths (part 3 of Supplementary Video 3). Therefore, the presence of nystagmus evoked in the lateral position of MCP suggests a distal location of the debris in the anterior canal of the uppermost ear.

This consideration can help us in the following ways. If we know the side of canal, we can tilt the head by 30° and make the Yacovino maneuver more effective. It will definitely bring down the number of Yacovino maneuvers performed in each patient. If we know that the left anterior canal is involved then we can ask the patient to sleep in the right lateral position for two days, which will help the particles go toward the common crus and eventually toward the utricle. We see the possibility of ourselves or other workers in trying the prolonged lateral decubitus position combined with the Yacovino maneuver for at least a subset of patients with positional down beating nystagmus.

### Reversal of Nystagmus

In our study, two subjects (case five and nine) had a reversal of nystagmus on getting up, which is an unusual but not new finding. Imbaud et al. suggested that reversal is seen in cases of AC BPPV after making the patient sit up from a supine position (27). Some authors contest this and argue that reversal of nystagmus is not seen in cases of AC BPPV (18, 19).

### Treatment

We performed the Yacovino maneuver in every patient and the definitive diagnosis of AC BPPV was made only after successful resolution of vertigo by the Yacovino maneuver.

If the side of involvement is known, the modified Yacovino maneuver is performed with the head turned 30° away from the affected side. The patient sits up at the end while maintaining 30° of head rotation.

If the side of involvement is not known, the Yacovino maneuver is completed but is not dependent on the side of the canal (5, 21, 24). It treats AC BPPV irrespective of the side of canal involved.

Surgical management has also been described by Naples et al., with plugging of anterior semicircular canal which is to be reserved for refractory cases (28).

## Conclusion

We conclude that anterior canal BPPV is a rare but definite entity. It may not be apparent on positional testing the first time, so repeated testing may be needed with more than one maneuver. The most consistent diagnostic maneuver is SHH though there were patients in whom findings could only be elicited during DHP testing.

We recommend a testing protocol that includes DHP testing on both sides and SHH. MCP testing may also evoke vertigo and DBN with or without a torsional component. The utility of this finding in determining the side involved deserves further exploration. Reversal of nystagmus during the reversal of testing position is unusual but can occur. The Yacovino maneuver is effective in resolving AC BPPV though some patients require up to four repetitions, requiring multiple visits.

We proposed a hypothesis that explains why DHP testing is sensitive to AC BPPV on either side, whereas an MCP lateral position on one side is only sensitive to one-sided AC BPPV.

## Data Availability Statement

The original contributions presented in the study are included in the article/Supplementary Material, further inquiries can be directed to the corresponding author/s.

## Author Contributions

SD, PP, and AR conducted the experiments, analyzed and interpreted the data, and wrote the manuscript. VP, AB, and PN conducted the experiments and analyzed and interpreted the data. RN conducted the design and conceptualization of the study, interpretation of the data, and revised the manuscript. All authors contributed to the article and approved the submitted version.

## Conflict of Interest

The authors declare that the research was conducted in the absence of any commercial or financial relationships that could be construed as a potential conflict of interest.

## Supplementary Material

Supplementary Video 1 Case 11—Dix-Hallpike left showing down beating nystagmus.

Supplementary Video 2 Case 11—Dix-Hallpike right showing down beating torsional (upper pole beating to right side) nystagmus.

Supplementary Video 3 Explanation of three types of debris movement in right lateral position of McClure Pagnini. First, when the particles are at/near to the ampulla of the left (uppermost ear) anterior canal, no/minimal movement is seen in right lateral position (part 1 of this video). Second, when the particles are slightly away or distal from the ampulla (which might be due to DHP or SHH), we can see that the particles are moving toward the common crus (part 2 of this video). Third, when the particles are in the right (undermost ear) anterior canal in the right lateral position, there is minimal or no movement of otoliths (part 3 of this video).

## Abbreviations

AC: anterior canal
BPPV: benign paroxysmal positional vertigo
DHP: Dix-Hallpike
DBN: down beating nystagmus
MCP: McClure Pagnini
SHH: supine head hanging
LC: lateral canal
PC: posterior canal
VNG: videonystagmography.
